# Improved marine predators algorithm for engineering design optimization problems

**DOI:** 10.1038/s41598-024-63826-x

**Published:** 2024-06-06

**Authors:** Ye Chun, Xu Hua, Chen Qi, Ye Xin Yao

**Affiliations:** 1 Internet of Things Engineering College, Jiangsu Vocational College of Information Technology, Wuxi, 214001 China; 2 School of Artificial Intelligence and Computer Science, Jiangnan University, Wuxi, 214001 China; 3 Institute of Civil Engineering, Jiangsu Vocational College of Information Technology, Wuxi, 214001 China; 4 Wuxi Furen High School, Wuxi, 214001 China

**Keywords:** Improved marine predators algorithm, Self-adaptive weight, Social strategy, Complex industrial engineering design problems, Engineering, Mathematics and computing

## Abstract

The Marine Predator Algorithm (MPA) has unique advantages as an important branch of population-based algorithms. However, it emerges more disadvantages gradually, such as traps to local optima, insufficient diversity, and premature convergence, when dealing with complex problems in practical industrial engineering design applications. In response to these limitations, this paper proposes a novel Improved Marine Predator Algorithm (IMPA). By introducing an adaptive weight adjustment strategy and a dynamic social learning mechanism, this study significantly improves the encounter frequency and efficiency between predators and preys in marine ecosystems. The performance of the IMPA was evaluated through benchmark functions, CEC2021 suite problems, and engineering design problems, including welded beam design, tension/compression spring design, pressure vessel design, and three-bar design. The results indicate that the IMPA has achieved significant success in the optimization process over other methods, exhibiting excellent performance in both solving optimal parameter solutions and optimizing objective function values. The IMPA performs well in terms of accuracy and robustness, which also proves its efficiency in successfully solving complex industrial engineering design problems.

## Introduction

Optimization problems are prevalent in scientific research and engineering applications such as financial management, energy scheduling, path planning, engineering design, and sensor network Optimization. These applications have drawn a great deal of attention from academics in related fields. In most scientific research as well as technical applications, the primary research direction is how to tackle optimization problems with the least amount of resources. Numerous new technologies are being developed and applied widely, which is leading to the continuous emergence of more complicated optimization challenges. Optimization issues have more variables, a wider solution scale, and more complexity than the prior single constraints. The complexity of the variable's dimensionality often increases exponentially for single-objective continuous optimization problems, multi-objective continuous optimization issues, and discrete optimization problems. To solve discrete optimization problems more effectively, the majority of currently available algorithms must be discretized because they are designed for continuous optimization, which makes it challenging to apply them directly to discrete optimization problems. As a result, to tackle optimization challenges, better algorithms must be created for each unique situation.

Optimization algorithms have garnered significant attention and in-depth study from researchers across fields very quickly. The majority of intelligent optimization algorithms are developed through animal simulations and the analysis of diverse creatures' communication patterns. The mayfly algorithm, which was proposed by Zervoudakis et al.^1^ and is inspired by the flight behavior and mating process of mayflies, is one of the novel representative algorithms. Mohammadi-Balani et al.^2^ proposed an algorithm based on the hunting spiral of a golden eagle by observing its speed in the golden eagle optimization algorithm is proposed by observing the way the golden eagle adjusts its speed at different stages of the trajectory. Yuan et al.^3^proposed a prevention-inspired bionic optimization algorithm, named Coronavirus Mask Protection Algorithm (CMPA) based on the virus transmission of COVID-19. In CMPA, the process of infection and immunity consists of three phases, including the infection stage, diffusion stage, and immune stage. Kaveh et al.^4^ simulated the territorial behavior of water striders, ripple communication, mating mode, feeding mechanism, and succession proposed the water strider algorithm. Chou et al.^5^ observed the pattern of jellyfish movements following the currents and in the jellyfish swarms, and by simulating the temporal control mechanism of switching between these movements as well as the process of converging into jellyfish blooms propose an artificial jellyfish search algorithm. Yuan et al.^6^ proposed a learning-imitation strategy-assisted alpine skiing optimization (LISASO). In LIS, the learning ability of individuals and the imitation of competitions are introduced to strengthen the association between individuals and the first individual. Yuan, YL; Ren, JJ[7]proposed a novel swarm intelligence optimization algorithm is proposed, which is named alpine skiing optimization (ASO). The main inspiration for the ASO originated from the behaviors of skiers competing for the championship.

The study and proposal of new algorithms with better performance are of great importance to the research of intelligent Optimization algorithms and the improvement of the theoretical system. The Marine Predator Algorithm (MPA) is a new intelligent Optimization algorithm proposed by Mirjalili et al.^8^ based on the foraging pattern of marine organisms. The Marine Predator Algorithm, which has the benefits of a simple structure, few parameters, and ease of implementation, solves the optimization problem by mimicking the laws of marine organisms foraging for prey, including sharks, toucans, sunfish, and swordfish. However, MPA presents weak points towards premature convergence, stuck into local optima, and lack of diversity, specifically, which is in the real-world niche problems within different industrial engineering design domains.

To get rid of such limitations, this paper presents an Improved Marine Predators Algorithm (IMPA) to mitigate above mentioned limitations by deploying the self-adaptive weight and dynamic social learning mechanism. IMPA can find an approximate optimal solution relatively quickly by combining dynamic social strategies and adaptive weight settings, and overcome limitations by avoiding local optimal solutions and improving overall performance. IMPA promotes balanced exploration and exploitation through dynamic social strategies and adaptive weights. The proposed method achieves enhanced solution quality, maintained population diversity, and accelerated convergence speed. 

## Background and related work

Since the traditional MPA was initially presented in 2020, several MPA versions have been put up for a wide range of optimization issues. Binary, discrete, modified, hybridized, chaotic, quantum and multi-objective MPA versions are the different classifications for these MPA variations. Abdel-Basset et al.^9^ created the IMPA to solve multi-threshold medical image segmentation. This approach improves the particles that fail to find a workable solution after a certain number of iterations by adding a ranking-based diversity reduction (RDR) mechanism to the traditional MPA. To detect COVID-19, the model utilized in this study operates on chest X-ray pictures. The experiment findings demonstrate that the IMPA provides higher-quality segmented images than other algorithms when compared against a wide range of other metaheuristics.

The IMPOA is suggested by Shaheen et al.^10^ as a solution to the CHP issue. By improving predator techniques that take environmental and climatic variation into account, IMPOA maintains a balance between the exploitation and exploration phases. Several limitations are taken into consideration when solving the CHP issue using the suggested technique. Test systems including 5, 48, 84, and 96 units were used to assess the IMPOA's efficacy. The simulation's findings demonstrate the IMPOA's superiority in terms of stability and pace of convergence.

In Yu et^11^, an adaptive MPA (AMPA) is presented as a way to minimize load demands by optimizing the configuration of a hybrid power system integrating batteries, PV, and diesel generator. Three objective functions, including the annualized cost, the value of CO2 emissions, and the chance of load loss for the hybrid power generation system, are minimized in this multi-objective optimization problem. The AMPA is compared with LOA, FOA, COA, and conventional MPA and validated on multiple benchmark functions. The simulation findings validate AMPA's best accuracy and fastest convergence. Shaheen, El-Sehiemy, et al.^12^ offer an improved MPA to address simultaneous distribution reconfiguration with distribution generations. The suggested method accounts for differences in the temperature and environment. The prey's new positions are updated in EMPA using a random probability. The voltage stability index (VSI) is improved and power losses are minimized using the suggested algorithm under different loading scenarios. EMPA's performance was evaluated using IEEE 33, 83, and 137-bus distribution networks. Compared to other competing algorithms, the EMPA produces better results by efficiently minimizing the problem under consideration.

In Yang et al.^13^, multi-strategy marine predators algorithm-joint regularized is a semi-supervised classification model. Machine Learning with Applications semi-supervised extreme learning machine (MSMPA) is built. MSMPA incorporates supervised information regularization and is based on Hessian regularization. MSMPA uses several techniques to enhance its functionality. To create a high-quality initial population for the MSMPA, a chaotic opposition learning strategy is applied during initialization. In each of the three stages of the MSMPA, adaptive inertia weights and adaptive step control factors are used to improve local optimal prevention, convergence speed, and exploration and exploitation capabilities. The simulation studies show that MSMPA exhibits better classification accuracy and stability than other competitive classification approaches.

In Houssein, Hassaballah et al.^14^, a novel nonlinear step factor control method is utilized to balance the exploration and exploitation stages, increase the pace of convergence, and improve the MPA's capacity for global search. Convolutional Neural Networks (CNNs) and the suggested IMPA are combined for the categorization of electrocardiograms (ECGs). The resulting model, dubbed IMPA-CNN, is tested on the European ST-T database, the St. Peters-burg INCART database, and the MIT-BIH arrhythmia database. The experimental results indicate the superiority of the IMPA-CNN models compared to MPA, GSA-NN, EO-CNN, HHOCNN, SCA-CNN, PSO-CNN, and WOA-CNN models using multiple assessment metrics.

In Liu et al.^15^, the MPA is combined with a novel predator encoding mechanism based on internet protocol (IP) to create an IPMPA algorithm. A deep convolutional neural network (DCNN) and the suggested IPMPA are merged to create a new model called DCNN-IPMPA, which is used for COVID-19 diagnosis. We evaluate the DCNN-IPMPA's performance using the COVID-CT and SARS-CoV-2 datasets. The outcomes of the simulation demonstrate that the DCNN-IPMPA model outperforms other models in terms of results.

Yuan et al.^16^ presented a novel assisted optimization strategy, named elite opposition-based learning and chaotic k-best gravitational search strategy (EOCS), which is proposed for the grey wolf optimizer (GWO) algorithm. In the EOCS-based grey wolf optimizer (EOCSGWO) algorithm, the elite opposition-based learning strategy (EOBLS) is proposed to take full advantage of better-performing particles for optimization in the next generations.

Aydemir, S. B[17]proposed a dynamic selection strategy named as FOCLMPA that adapts during the evolutionary process. This dynamic approach assigns higher selection probabilities to parents with superior fitness, resulting in accelerated convergence and heightened exploration capabilities. They innovatively suggested a dynamic dimension and a greedy strategy by dimension approach. This strategy evaluates solutions in each dimension, mitigating the risk of local optima, thereby enhancing the overall performance of the algorithm.

Du and Guo^18^ventured into hybridization named as EMPA by combining the Marine Predators Algorithm with opposition-based learning. This hybrid approach combines opposing initial numbers with a self-adaptive component strategy, effectively liberating the algorithm from local optima traps and facilitating superior convergence. These include opposition learning to expand the search range, adaptive evolution for heightened global exploration, neighborhood search to diversify the population, and greedy selection to ensure solution quality.

Han and Du^19^ presented a modification to the MPA algorithm by adjusting conversion parameters. This adjustment transitions from a linear decline to a nonlinear one, optimizing the balance between global and local exploration. They put forward a hybrid algorithm seamlessly integrating the exploitation capabilities of crossover with individual solutions' personal best states, self-learning mechanisms, and global search mechanisms. This algorithm updates solutions using sine or cosine strategies, implementing a novel approach to mutualism phases.

Kumar et al.^20^ have demonstrated the remarkable prowess of MPA in addressing multi-objective problems related to real-time task scheduling within multiprocessor systems, underscoring its relevance in the realm of computational optimization.

Nonetheless, like so many other metaheuristics, the MPA exhibits some disadvantages due to its numerous decision variables, dense local optimal solutions, and high computational effort. The Marine Predator Algorithm will converge to the local optimal position in the space, stretching from the current number to the opposite number which has made less progress in handling high-dimensional optimization problems than the majority of the aforementioned research. For this purpose, The processes for the development of the Improved Marine Predators Algorithm (IMPA) with self-adaptive weight and dynamic social strategy have been performed. The motivation for this article is listed below.

1. Self-adaptive weight parameter tuning scheme is adopted into the IMPA. according to the proportion of fitness value;
2. Dynamic Social Mechanism was implemented to balance exploration and exploitation, preventing premature convergence to a local optimal position;
3. The proposed algorithm was tested by 23 benchmark test functions and IEEE CEC 2021 benchmark, compared to state-of-the-art algorithms and different MPA variants.
4. The proposed algorithm was tested on four different real-world engineering problems and compared with some algorithm in the literature.

The remaining part of this paper is organized as follows. Section "Marine Predators Algorithm" describes the Marine Predators Algorithm. Section "Improved Marine Predators Algorithm" describes the Improved Marine Predators Algorithm in detail. Section "Experiments and discussion" gives the experimental results and analysis of the benchmark function. Section “Engineering design optimization” examined the IMPA in engineering problems. Section “Conclusion” concludes this paper and indicates future research.

## Marine predators algorithm

The Marine Predators Algorithm (MPA) operates as a population-based algorithm, a characteristic shared with other metaheuristic algorithms. One of its distinctive features involves periodically disseminating the initial solution across the search space for initial demonstration purposes, as expressed in the following equation in Eq. (1):

1 $$X_{0} = X_{\min } + rand(X_{\max } - X_{\min } )$$

Rand represents a random vector between 0 and 1, while $X_{\min }$ and $X_{\max }$ denote the lower and upper bounds of the parameters being optimized. This initial dispersion of solutions sets the stage for subsequent optimization steps. In the context of MPA, the best solution discovered is identified as the dominant Predators, and it plays a pivotal role in the creation of a matrix known as *Elite*, as depicted in Eq. (2). These matrices are instrumental in tracking and locating prey, relying on the positional information of the prey itself.

2 $$Elite = \left[ \begin{gathered} X_{1,1}^{I} ,X_{1,2}^{I} ,...X_{1,d}^{I} \hfill \\ X_{2,1}^{I} ,X_{2,2}^{I} ,...X_{2,d}^{I} \hfill \\ ............. \hfill \\ X_{n,1}^{I} ,X_{n,2}^{I} ,...X_{n,d}^{I} \hfill \\ \end{gathered} \right]_{n \times d}$$

For the *Elite* matrix, $X_{n,d}^{I}$ represents the best predators vector, where *n* corresponds to the number of Predators, and *d* is the number of dimensions. The *Elite* matrix undergoes updates when a superior agent replaces the best predators. The *Prey* matrix, on the other hand, shares a similar dimensionality with the *Elite* matrix. Agents in pursuit of prey adjust their positions based on the information contained within the *Prey* matrix. Therefore, the initialization phase is pivotal in generating the initial *Prey* matrix, with the best predators being responsible for creating the *Elite* matrix. Equation (3) provides an expression for the *Prey* matrix.

3 $$\Pr ey = \left[ \begin{gathered} X_{1,1} ,X_{1,2} ,...X_{1,d} \hfill \\ X_{2,1} ,X_{2,2} ,...X_{2,d} \hfill \\ ............. \hfill \\ X_{n,1} ,X_{n,2} ,...X_{n,d} \hfill \\ \end{gathered} \right]_{n \times d}$$

In essence, the entire optimization process in MPA hinges on the interactions and dynamics governed by the *Prey* and *Elite* matrices, encapsulating the algorithm's unique approach to problem-solving. There are three phases in the MPA’s optimization process:

Phase 1—High Velocity Ratio (HVR): This initial phase occurs when the prey's speed falls below that of the predators, emphasizing the importance of exploration in the early stages of optimization. The mathematical representation of this phase is given by in Eq. (4):

*While Iteration* < *1/3* × *max(Iteration)*

4 $$\begin{gathered} stepsize_{i} = R_{B} \otimes (Elite_{i} - R_{B} \otimes \Pr ey_{i} ),i = 1,...,n \hfill \\ \Pr ey_{i} = \Pr ey_{i} + P \cdot R \otimes stepsize_{i} \hfill \\ \end{gathered}$$

$R_{B}$ represents the Brownian Motion utilizing a regularly distributed random vector. The symbol ⊗ denotes entry-wise multiplications, simulating the prey's motion via the multiplication of $R_{B}$. The phase introduces two variables: a constant number *P* and a vector of random values *R* ranging from 0 to 1.

Phase 2—Uniform Velocity Ratio (UVR): In this phase, both prey and predators move at comparable speeds, necessitating a balance between exploration and exploitation. Half of the agents are assigned to exploration, and the other half to exploitation, with both predators and prey sharing these responsibilities. The mathematical representation during this phase is as follows:

*While 1/3* × *max(Iteration)* < *Iteration* < *2/3* × *max(Iteration)*

The first half of searching agents in Eq. (5)

5 $$\begin{aligned} stepsize_{i} & = R_{L} \otimes (Elite_{i} - R_{L} \otimes \Pr ey_{i} ),i = 1,...,n/2 \\ \Pr ey_{i} & = \Pr ey_{i} + P \cdot R \otimes stepsize_{i} \\ \end{aligned}$$

$R_{L}$ is a randomly generated vector based on Levy Flight (LF). It simulates prey movement by adding prey location to the step size. The MPA posits that the other 50% of the population follows the rules outlined in Eq. (6).

6 $$\begin{aligned} stepsize_{i} & = R_{B} \otimes (R_{B} \otimes Elite_{i} - \Pr ey_{i} ),i = n/2,...,n \\ \Pr ey_{i} & = Elite_{i} + P \cdot CF \otimes stepsize_{i} \\ CF & = (1 - \frac{Iteration}{{Max(Iter)}})^{{2 \times \frac{Iteration}{{Max(Iter)}}}} \\ \end{aligned}$$

Phase 3—Low Velocity Ratio (LVR): This stage occurs when the prey moves more slowly than the predators and is defined as in Eq. (7) :

*While Iteration* > *2/3* × *max(Iteration)*

7 $$\begin{aligned} stepsize_{i} & = R_{L} \otimes (R_{L} \otimes Elite_{i} - \Pr ey_{i} ),i = 1,...,n \\ \Pr ey_{i} & = Elite_{i} + P \cdot CF \otimes stepsize_{i} \\ CF & = (1 - \frac{Iteration}{{Max(Iter)}})^{{2 \times \frac{Iteration}{{Max(Iter)}}}} \\ \end{aligned}$$

Eddy formation or Fish Aggregation Devices (FADs), which can impact the algorithm's behavior. These factors are considered as local optima avoidance operators and influence the optimization process. Longer hops during simulation are considered to minimize stagnation in local optima. The impact of FADs is outlined as follows in Eq. (8):

8 $$\Pr ey_{i} = \left\{ \begin{gathered} \Pr ey_{i} + CF[(X_{\max } - X_{\min } ) \otimes R + X_{\min } ] \otimes U,if,r \le FADs \hfill \\ \Pr ey_{i} + (\Pr ey_{i} - \Pr ey_{i} )[(1 - r)FADs + r],if,r > FADs \hfill \\ \end{gathered} \right.$$

**Figure 1:**
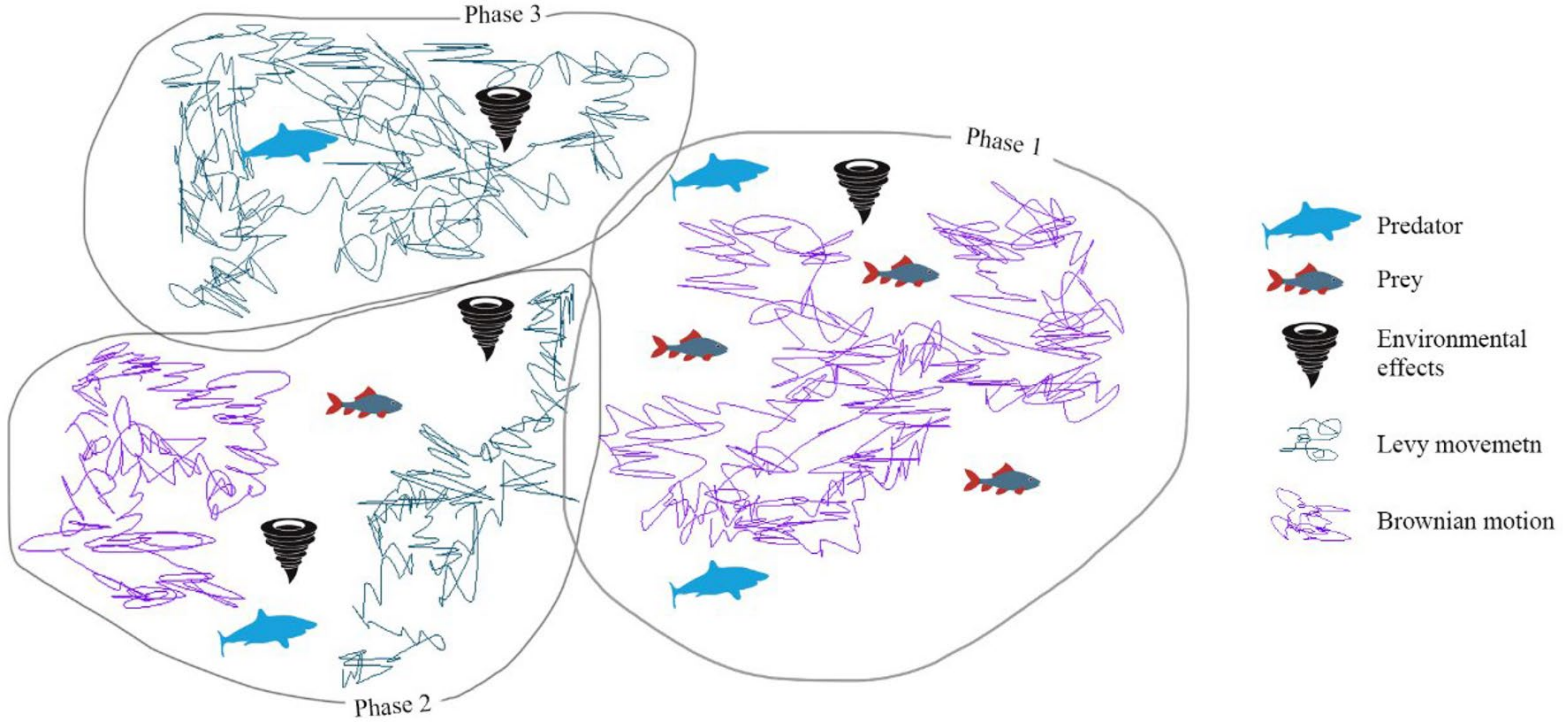
Marine Predators Algorithm.

These phases and adaptive strategies define the dynamics of the MPA, which is presented in Fig. 1. They are enabling it to navigate the optimization landscape efficiently while addressing different scenarios and challenges.

## Improved Marine Predators Algorithm

### Self-adaptive Weight

In this paper, we provide a novel optimization method that includes an adaptive weight parameter in the process. By harnessing the adaptive nature of this parameter, our approach enhances the algorithm's global exploration capability and its ability to escape local optima, all while maintaining strong performance in local refinement when optimization conditions stabilize. Specifically, during the initial stages of optimization, our approach accelerates global exploration of the solution space. As the optimization process stabilizes, it shifts focus toward local solution development, achieving a balance between global and local exploration. To achieve this, we leverage the positional information of the destination point through the design of a self-adaptive weight position-update mechanism, denoted as Eq. (9)-Eq. (12):

*While Iteration* < *1/3* × *max(Iteration)*

9 $$\Pr ey_{i} = \omega \cdot \Pr ey_{i} + P \cdot R \otimes stepsize_{i} ,\quad i = 1,...,n$$

*While 1/3*×*max(Iteration)*<*Iteration*<*2/3*×*max(Iteration)*

10 $$\Pr ey_{i} = \left\{ \begin{gathered} \omega \cdot \Pr ey_{i} + P \cdot R \otimes stepsize_{i} ,\quad i = 1,...,n/2 \hfill \\ \omega \cdot Elite_{i} + P \cdot CF \otimes stepsize_{i} ,\quad i = n/2,...,n \hfill \\ \end{gathered} \right.$$

*While Iteration* > *2/3* × *max(Iteration)*

11 $$\Pr ey_{i} = \omega \cdot Elite_{i} + P \cdot CF \otimes stepsize_{i} \quad i = 1,...,n$$

12 $$\omega = (\omega_{init} - \omega_{end} ) \times R \times \frac{{(n - i)\Pr ey_{i} }}{{nElite_{i} }},\quad \omega_{init} = 0.9,\omega_{end} = 0.4$$

It is difficult to tune the model and find a suitable weight parameter, *ω*. We want to minimize the function of our model by changing the weight parameters. Bayesian optimization helps us to find the most suitable point for the weighting parameter in the fewest number of steps. Bayesian optimization also uses an Acquisition Function^21^, which directs the sampling to regions that are likely to be better than the current best observation. The Acquisition Function, brought into the model, picks the best performing parameters. The tuning is cross-validated by the Grid Search CV^22^, which iterates through all permutations of the incoming parameters and returns the evaluation metrics scores for all parameter combinations by means of cross-validation.

Our enhanced approach replaces premature convergence, which improves the subpar search capability observed. The weight parameter, denoted as* ω*, is a self-adaptive balancing factor. In this study, we propose a self-adaptive weight *ω* that guides dynamic correction within the update formula, where $\Pr ey_{i}$ represents the best fitness value of the current iteration for the position variable and $Elite_{i}$ signifies the global optimal fitness value. Its function is to dynamically adjust the weight between the destination point and the current individual's position.

When the fitness value of the current position variable surpasses the global fitness value, the inertia weight assumes a higher value, which enhances global exploration capabilities and expands the search space for feasible solutions. Conversely, the self-adaptive weight generates a smaller value, promoting faster convergence rates and facilitating local refinement. The self-adaptive weight plays a critical role in enabling particles within the MPA to autonomously select between global and local phases, thereby enhancing accuracy and reducing the likelihood of falling into local optima.

### Dynamic social strategy

In this section, we introduce another critical element of the Improved Algorithm, which balances the potential neighborhood information of Elite Predators with that of other individuals and employs it to enhance the MPA. This provides more effective updates for the optimal individuals, reducing the probability of falling into local optima and attempting to overcome premature convergence. This significantly increases the probability of the population reaching the global optimum, thereby enhancing the development capacity.

This dynamic social strategy optimizes the search space and accelerates the effectiveness of the search process in the IMPA. The modified search mechanism introduced in the IMPA is expressed as follows:

*While Iteration* < *1/3* × *max(Iteration)*

13 $$\Pr ey_{i} = \omega \cdot \Pr ey_{i} + P \cdot R \otimes stepsize_{i} + (Elite_{i} - \Pr ey_{i} ),\quad i = 1,...,n$$

*While 1/3* × *max(Iteration)* < *Iteration* < *2/3* × *max(Iteration)*

14 $$\Pr ey_{i} = \left\{ \begin{gathered} \omega \cdot \Pr ey_{i} + P \cdot R \otimes stepsize_{i} + (Elite_{i} - \Pr ey_{i} ),\quad i = 1,...,n/2 \hfill \\ \omega \cdot Elite_{i} + P \cdot CF \otimes stepsize_{i} + (Elite_{i} - \Pr ey_{i} ),\quad i = n/2,...,n \hfill \\ \end{gathered} \right.$$

*While Iteration* > *2/3* × *max(Iteration)*

15 $$\Pr ey_{i} = \omega \cdot Elite_{i} + P \cdot CF \otimes stepsize_{i} + (Elite_{i} - \Pr ey_{i} ),\quad i = 1,...,n$$

In this optimization methodology, The second crucial component in the search process is the social component, denoted as $(Elite_{i} - \Pr ey_{i} )$, which improves the ability to develop solutions locally while still maintaining the ability to escape from local optima. When the search area provided by the coefficient is very large, the updated solution may diverge from the current state to avoid falling into local optima.

This dynamic social component amalgamates with the self-adaptive weight, collectively providing guidance to the current solution. By combining the directional influence of the best solution state and the best population state, the newly developed algorithm encompasses the following steps:

**Pseudocode:**
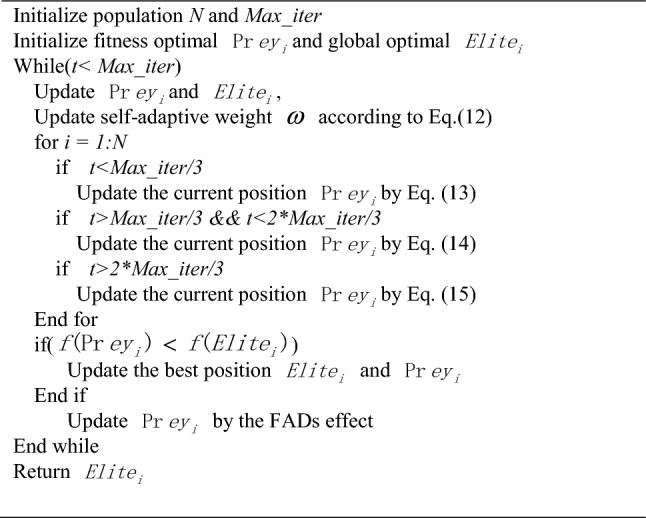
The Improved Marine Predators Algorithm.

As the parameters of the IMPA is concerned, N is the population , D is the dimension and *Max_iters* is Number of iterations. The time complexity of setting the adaptive weight and Dynamic Social Strategy is *O(N·D)*.The time complexity of IMPA is *O(N·D·Max_iter)*, which is the same as that of the origin MPA algorithm, indicating that the two improved strategies do not increase the computational burden of the Marine Predator Algorithm.

**Figure 2:**
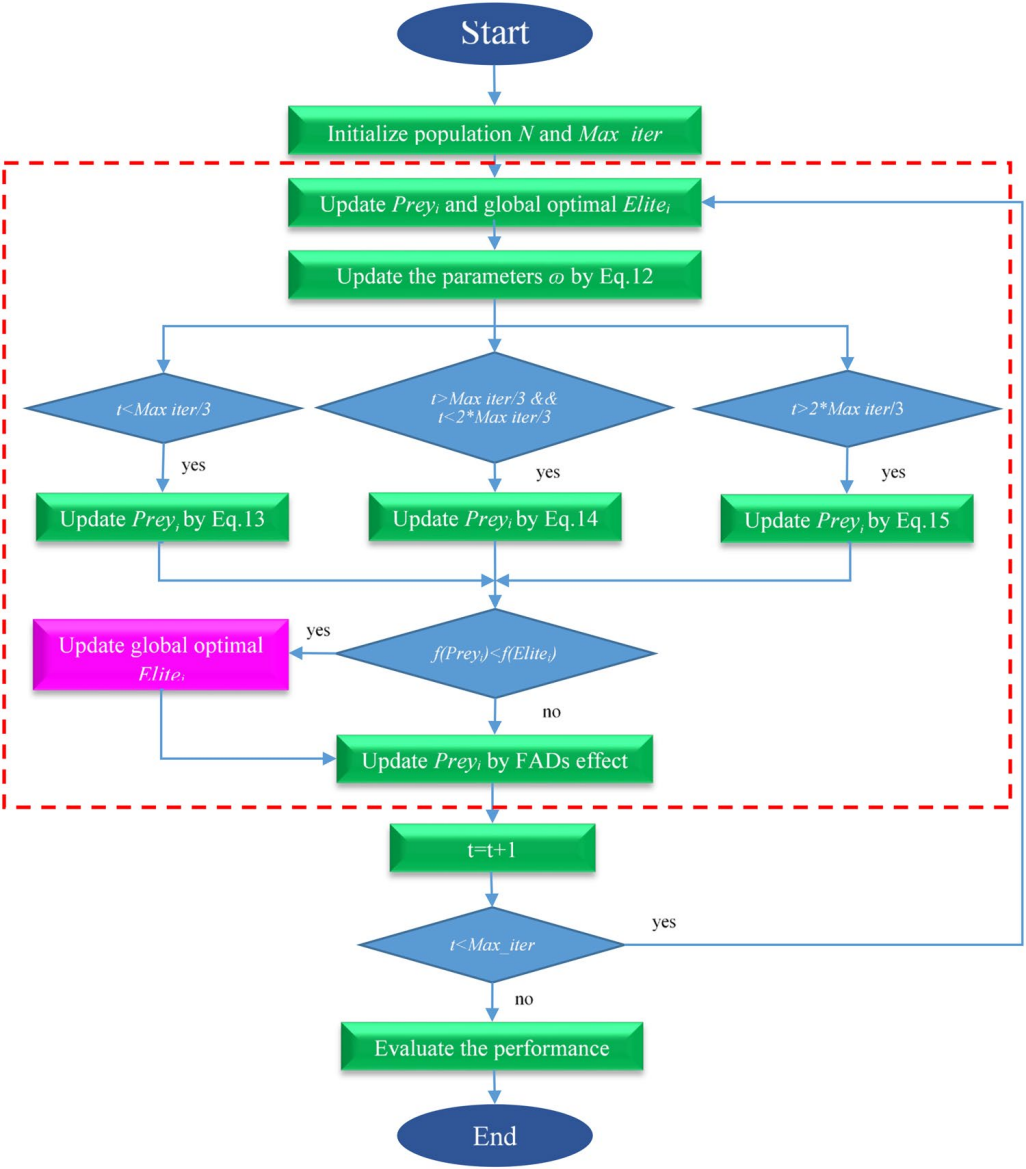
Framework of IMPA.

Our improved algorithm introduces a random selection mechanism for self-adaptive weight and dynamic social strategy, ensuring a harmonious balance between predators and prey position updates. Self-adaptive weight parameter tuning scheme is adopted into the IMPA, according to the proportion of fitness value. Dynamic social mechanism was implemented to balance exploration and exploitation, preventing premature convergence to a local optimal position. This comprehensive approach, which combines dynamic weight adaptation and social strategy, significantly enhances the optimization capabilities of the IMPA, improving its performance across a range of optimization problems. Thus the phase of opposition based weight and self-adaptive strategy provides an enhanced global and local search which helps in increasing appropriate diversity and avoids the skipping of true solutions. The flow of search process of proposed IMPA is presented in Fig. 2 A detailed analysis off enhanced diversity of solutions and exploitation of search space has been done in experimental section.

## Experiments and discussion

### Benchmark Problem Experiments

In the realm of optimization research, a fundamental aspect of assessing the effectiveness of newly proposed algorithms lies in the rigorous evaluation process employed. In this context, our study undertakes a comprehensive examination of the IMPA across a diverse range of benchmark problems. These benchmark functions, numbering 23 in total, are categorized into three distinct classes: unimodal, multimodal, and fixed-dimension problems^23,24^.

For the evaluation of these classical problems, a population size of 50 solutions was employed with a uniform termination criterion of 1000 iterations applied to all algorithms, whose dimensions are set up to 50. To ensure the robustness and reliability of our findings, a thorough comparative analysis was conducted based on two evaluation criteria: the mean and standard deviation. This evaluation was carried out through 20 independent runs on each benchmark function, yielding a comprehensive assessment of the algorithm's efficacy. Experimental computations were executed using MATLAB 2020a on a personal computer equipped with a 3.2 GHz CPU and 16 GB RAM.

The proposed method must show its competitive performance over some of those truly state-of-the-art methods. The results of our study reveal that the IMPA exhibits superior optimization performance compared to the MPA and several of the most recently proposed state-of-the-art methods algorithms, including Rime Optimization Algorithm (RIME)^25^, African Vultures Optimization Algorithm (AVOA)^26^, Artificial Gorilla Troops Optimizer (GTO)^27^, Optical Microscope Algorithm (OMA)^28^and Improved Sine Cosine Algorithm (IWSCA)^29^. Furthermore, this section includes a comparative analysis with some improved versions of MPA, specifically Enhanced MPA (EMPA)^30^ and Gradient-Descent-based MPA (GDMPA)^31^. The detailed results of this comparative assessment are presented in Table 1.

**Table 1:** Comparison results for benchmark functions.

		RIME	AVOA	GTO	OMA	IWSCA	MPA	EMPA	GDMPA	IMPA
F1	Mean	1.83E+00	0*	0*	73,711.65976	2.0325E−305	1.10586E−45	9.551725	73,926.97	0*
	Std	0.033687241	0*	0*	103,280.8621	0	1.47981E−45	12.23421	17,189.45	0*
F2	Mean	2.48E+00	0*	0*	8.22018E+30	5.0237E−149	7.15291E−27	1.659189	2.35E+13	0*
	Std	0.177327304	0*	0*	1.16251E+31	6.3254E−149	6.43065E−27	1.124542	3.01E+13	0*
F3	Mean	4.82E+03	0*	0*	311,140.7274	3.12E−285	4.62262E−08	16,277.87	101,277.1	0*
	Std	1420.11283	0*	0*	437,620.7511	0	6.4216E−08	2627.994	15,784.57	0*
F4	Mean	1.73E+01	0*	0*	74.25635165	1.6394E−157	1.61174E−17	0.311318	69.86455	0*
	Std	0.348893943	0*	0*	36.30472632	2.3185E−157	1.74054E−18	0.10907	1.301586	0*
F5	Mean	3.13E+02	2.79E−05*	43.92575	316,890,837.4	4.81765E−05	43.1203333	133.2982	2.84E+08	2.29769E−05
	Std	74.50977091	3.88E−05*	0.305613	448,018,563	6.62317E−05	0.085618377	119.5431	10,878,301	2.46087E−05
F6	Mean	2.40E+00	2.13E−08	0.037744	79,512.904	0.04510626	2.62536E−08	1.900509	78,735.79	7.63259E−10*
	Std	1.516431193	2.91E−09	0.045125	111,396.0966	0.034909693	6.15778E−09	0.583362	3349.898	1.06888E−09*
F7	Mean	3.87E−02	1.13E−05	2.13251E−05	246.203361	0.000117546	0.001040433	0.100476	180.8921	4.35E−06*
	Std	0.011201032	9.32E−06	8.09298E−06	346.3149937	0.000148561	0.000655853	0.0712	0.19872	6.05E−06*
F8	Mean	−1.72E+04	−13,021.3	−20,949.14436	−3712.911898	−7174.099901	−15,551.89182	−11,583.9	−7865.45	−2.09E+04*
	Std	24.60148659	242.4315	3.49852E−10	5026.223273	249.3668736	191.9082888	371.0039	671.9733	4.26E−07*
F9	Mean	0*	3.04E+01	0*	0*	1007.907608	0*	69.13439	599.6754	0*
	Std	0*	4.99079528	0*	0*	72.52931792	0*	19.03083	56.75393	0*
F10	Mean	1.91E+00	8.88E−16	8.88178E−16	16.33314408	8.88178E−16	4.44089E−15	1.6573	17.78133	8.88E−16*
	Std	0.286164829	0	0	6.902385394	0	0	1.403677	0.316152	0*
F11	Mean	8.12E−01	0*	0*	882.3145682	0*	0*	0.081999	391.6581	0*
	Std	0.002075913	0*	0*	1243.775962	0*	0*	0.094563	11.37449	0*
F12	Mean	5.38E+00	4.68E−10	0.022478	721,570,701.2	0.001656308	2.0154E−09	0.015901	5.36E+08	5.52733E−11*
	Std	1.759051378	3.13E−10	0.012132	1,020,454,988	0.00094689	1.06642E−10	0.001225	21,710,845	7.00712E−11*
F13	Mean	4.97E−01	3.88E−09	0.117997	1,422,848,975	4.38298E−07	2.7927E−08	0.051358	1.04E+09	2.12751E−09*
	Std	0.214264812	3.44E−10	0.160096	2,012,158,438	6.14402E−07	6.97689E−09	0.030578	13,698,206	2.90836E−09*
F14	Mean	3.46E+00	9.98E−01	3.950756354	261.9782921	7.951564594	0.998003838	4.507942	6.163716	0.998004*
	Std	3.486631297	4.71E−16	4.175822655	336.6126789	6.67359067	0	2.134801	2.461043	0*
F15	Mean	1.10E−02	4.00E−04	0.000542792	0.959327936	0.002699257	0.000307486	0.002224	0.001735	0.000307*
	Std	0.013212518	5.78E−05	0.00017189	1.324822465	0.002464541	3.83323E−19	0.00033	0.000633	7.67E−20*
F16	Mean	−1.03E+00	−1.03E+00	−1.031248995	321.9470001	−0.984958941	−1.031628453	−1.01995	−1.02442	−1.03163*
	Std	1.02809E−07	2.23E−12	0.000487899	455.6062817	0.063957969	0	0.006994	0.009946	0*
F17	Mean	3.98E−01	3.98E−01	0.497658429	4.200093154	0.402981939	0.397887358	0.437015	0.413355	0.397887*
	Std	3.56092E−05	1.26E−15	0.138064149	5.342065731	0.005560347	0	0.017909	0.021466	0*
F18	Mean	4.61E+01	1.65E+01	17.60271856	115.6570207	25.20217481	3	3.300957	3.536866	3*
	Std	60.95042098	1.91E+01	20.63516026	112.6090904	7.155485643	0	0.13192	0.496593	0*
F19	Mean	−3.86E+00	−3.86E+00	−3.862772367	−3.666673049	−3.834087963	−3.862782148	−3.85669	−3.82952	−3.86278*
	Std	9.14435E−07	1.61E−05	1.35031E−05	0.2766239	0.004224394	0	0.000615	0.027548	0*
F20	Mean	−3.32E+00	−3.26E+00	−3.322	−1.887759676	−3.064140909	−3.321995172	−2.95493	−2.90161	−3.262548611*
	Std	1.01605E−05	8.44E−02	2.51E−15	2.027932031	0.0644847	2.22045E−15	0.102336	0.012301	0.084070132*
F21	Mean	−3.87E+00	−1.02E+01	−10.15319967	−3.20572885	−10.10327247	−10.15319968	−7.15709	−2.88735	−10.1532*
	Std	1.746735607	1.04E−07	6.67166E−09	4.367303045	0.055574157	1.77636E−15	3.322242	0.220971	1.78E−15*
F22	Mean	−1.04E+01	−1.04E+01	−10.40294057	−4.44037456	−10.36647304	−10.40294057	−4.65739	−4.27223	−10.4029*
	Std	0.001170383	3.19E−09	3.06354E−10	6.00658441	0.048483391	0	2.28222	0.029231	0*
F23	Mean	−1.05E+01	−8.67E−01	−1.05E+01	−10.53640979	−0.22686473	−10.52977601	−5.29647	−3.33951	−10.5364*
	Std	0.00E+00	0.228671286	7.95E−08	3.97204E−08	0.066386287	0.001101293	1.605098	0.205943	3.97E−15*

*Represent the optimal solution of the function.

In our comparative analysis, we examined the experimental results of various algorithms applied to the F1-F23 functions. The IMPA consistently attained the theoretical global optimum for 22 out of the 23 functions (F1-F4, F6-F23). Among the F1-F4 functions, the improved algorithm AVOA, and GTO algorithm can obtain the optimal solution, but the IMPA has better experimental results in other functions except for F5. The experiment shows that the improved algorithm performs better results in the remaining results.

The comparative performance of RIME, OMA, and IWSCA fell short when compared to the exceptional performance of the IMPA across all 23 benchmark functions. Notably, the IMPA surpassed other improved versions of MPA, such as EMPA and GDMPA. These findings underscore IMPA's superior performance in terms of global exploration capabilities and its ability to evade local optima in comparison to the other algorithms.

**Figure 3:**
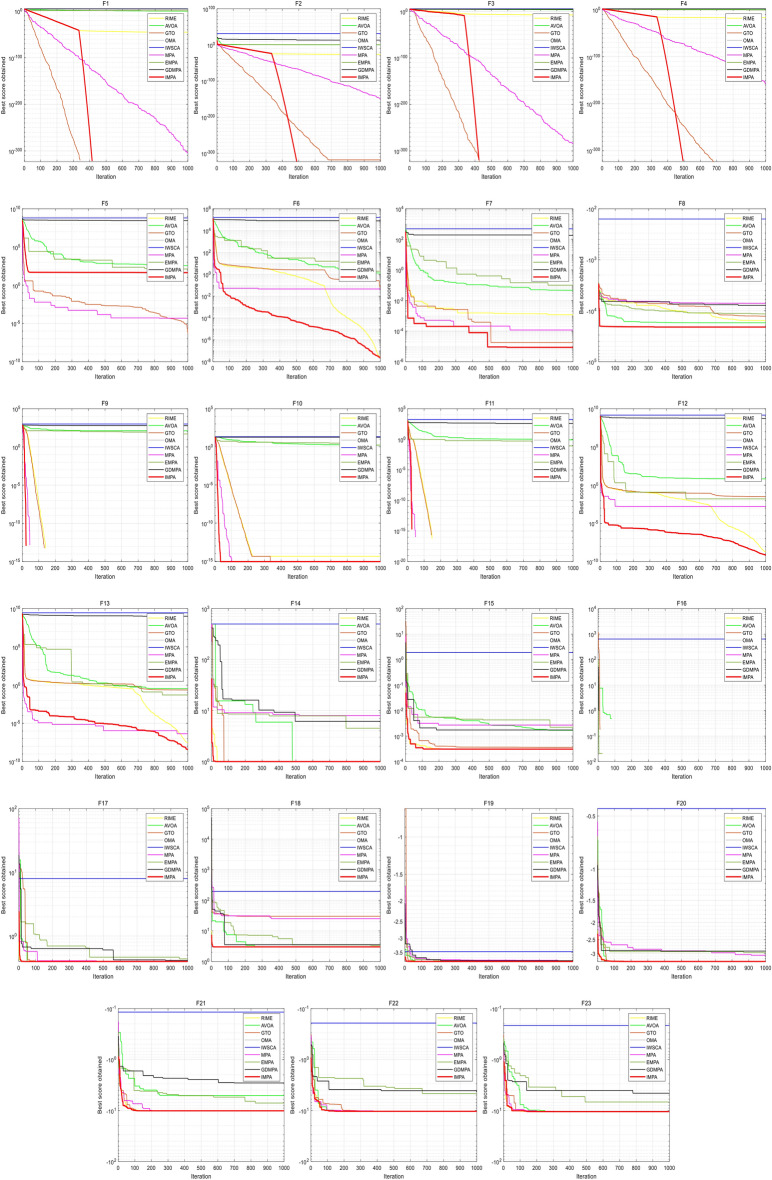
Convergence curves for benchmark functions.

This study additionally presents the convergence curves for the nine optimization algorithms refer to Fig. 3. The results indicate that the IMPA excels in reaching the theoretical global minimum for the Unimodal functions (F2-F4), achieving this within approximately 400 to 500 iterations. For the Multimodal functions (F9-F11), the IMPA also exhibits faster convergence than other algorithms, reaching the theoretical global minimum in 100 iterations. Even in the context of fixed-dimension problems, the IMPA demonstrates superior convergence speed and accuracy relative to other algorithms, highlighting its distinctive characteristics.

Conversely, the other algorithms consistently experience premature convergence across all benchmark functions, yielding solutions consistently inferior to those of IMPA. Although MPA and GTO perform better than IMPA in only two functions (F1 and F5), they lag behind in other scenarios. The convergence curves clearly demonstrate that IMPA consistently reaches the theoretical global minimum for most benchmark functions, underscoring its superior optimization performance. The outstanding convergence speed of IMPA significantly reduces the computational complexity of the optimization problem.

This study additionally presents the convergence curves for the nine optimization algorithms refer to Fig. 3. The results clearly indicate that the IMPA excels in reaching the theoretical global minimum for the Unimodal functions (F2-F4), achieving this within approximately 400 to 500 iterations. For the Multimodal functions (F9-F11), the IMPA also exhibits faster convergence than other algorithms, reaching the theoretical global minimum in 100 iterations. Even in the context of fixed-dimension problems, the IMPA demonstrates superior convergence speed and accuracy relative to other algorithms, highlighting its distinctive characteristics.

Conversely, the other algorithms consistently experience premature convergence across all benchmark functions, yielding solutions consistently inferior to those of IMPA. Although MPA and GTO perform better than IMPA in only two functions (F1 and F5), they lag behind in other scenarios. The convergence curves clearly demonstrate that IMPA consistently reaches the theoretical global minimum for most benchmark functions, underscoring its superior optimization performance. The outstanding convergence speed of IMPA significantly reduces the computational complexity of the optimization problem.

In order to illustrate whether there are differences between the IMPA and the comparison algorithm, Wilcoxon rank-sum test^23^ and Friedman test are also introduced to analyze the results of the algorithm, which are presented in Tables 2 and 3. The significance *p-value* is set to 0.05, When the *p-value* in the Wilcoxon rank-sum test is less than 0.05, it means that there is a significant difference between the algorithm proposed in this article and the comparison algorithm. Otherwise, it means that there is no significant difference between the algorithm in this article and the comparison algorithm. In the result table, ‘+’ denotes the IMPA is better than another counterpart, ’ -’ means other algorithms are better, and ’NAN’ means there is no significant difference between the two algorithms after the test. The results show that the *p-value* of the OMA and the GTO on the function is greater than 0.05, indicating that there is no significant difference between the two algorithms and the IMPA. In addition, the *p-value* of the IMPA is less than 0.05, indicating that there is a significant difference between the IMPA and other comparison algorithms. it is obvious that the IMPA performs better than other algorithms, and the improvement strategy introduced in the IMPA is effective.

**Table 2:** Wilcoxon rank-sum test.

	IMPA																						
	F1	F2	F3	F4	F5	F6	F7	F8	F9	F10	F11	F12	F13	F14	F15	F16	F17	F18	F19	F20	F21	F22	F23
RIME	+	+	+	+	+	NAN	+	+	+	+	+	+	+	+	+	+	NAN	+	+	+	+	+	+
AVOA	+	+	+	+	+	+	+	+	+	+	NAN	+	+	+	+	+	+	+	+	+	+	+	+
GTO	−	−	−	−	NAN	−	−	−	−	−	−	−	−	−	−	NAN	−	−	−	−	−	−	−
OMA	−	−	−	−	−	−	−	−	−	−	−	−	−	−	−	−	NAN	−	−	−	−	−	−
IWSCA	+	+	+	+	+	+	+	+	+	+	+	+	+	+	+	+	+	+	+	+	+	+	+
MPA	+	+	+	+	+	+	+	+	+	+	+	+	+	+	+	+	+	+	NAN	+	+	+	+
EMPA	+	+	+	+	+	+	+	+	+	+	+	NAN	+	+	+	+	+	+	+	+	+	+	+
GDMPA	+	+	+	+	+	+	+	+	+	+	+	+	+	+	+	+	+	+	+	+	+	+	+

**Table 3:** Friedman test.

	**IMPA**	**RIME**	**AVOA**	**GTO**	**OMA**	**IWSCA**	**MPA**	**EMPA**	**GDMPA**
F1	1.50*	5.00	10.00	1.50	6.00	8.97	3.00	4.00	7.00
F2	1.00*	5.00	9.97	2.00	6.00	7.00	3.00	4.00	8.00
F3	1.50*	3.83	6.83	1.50	5.00	7.67	10.00	3.17	9.00
F4	1.00*	4.23	8.03	2.00	4.77	9.27	9.03	3.00	6.43
F5	1.77	6.77	8.13	1.23	8.10	7.90	4.70	4.60	5.40*
F6	2.50*	2.50	8.00	6.97	2.50	5.97	9.00	10.00	2.50
F7	1.50*	2.40	8.07	2.33	5.03	8.93	4.70	5.07	10.00
F8	1.00*	6.83	8.23	2.97	4.83	8.77	2.03	4.50	5.83
F9	3.28*	3.28*	7.63	3.28*	5.20	9.60	3.28*	3.47	7.10
F10	1.60*	3.87	9.50	1.60	9.50	8.00	3.30	4.63	6.00
F11	3.43*	3.43	8.60	3.43	3.43	7.60	3.80	3.70	10.00
F12	3.63*	5.60	9.40	3.10	8.03	9.57	5.17	6.83	1.60*
F13	3.80	6.93	7.87	3.23	9.77	4.55	6.57	8.40	1.00*
F14	5.82	7.20	2.95*	3.12	6.80	2.95*	5.13	7.07	9.20
F15	4.30	6.23	2.43*	4.73	5.20	8.20	7.23	3.57	9.90
F16	4.13*	9.00	4.13*	4.13*	4.13*	4.13*	4.13*	7.07	4.13*
F17	7.02	8.93	3.07*	4.62	9.90	3.07	5.17	7.10	3.07*
F18	3.33*	8.83	3.33*	4.45	9.93	3.33*	7.32	7.80	3.33*
F19	2.70	7.30	2.70	7.90	9.60	2.70	8.50	6.60	4.30
F20	1.88*	6.50	2.83*	7.90	9.60	5.45	6.77	5.13	6.20
F21	5.35	8.23	3.77*	7.53	8.97	4.22	5.03	3.93	4.70
F22	2.60*	8.63	2.60*	8.20	9.03	3.42	6.53	5.03	5.87
F23	2.17*	8.70	2.17*	7.90	9.20	3.33	6.10	3.93	4.93
Mean	3.21*	6.05	6.10	4.16	6.98	6.29	5.63	5.33	5.40
Std	1.69*	2.13	2.83	2.34	2.35	2.44	2.14	1.85	2.91

*Represent the optimal solution of the function.

In Table 3, Friedman test is used to verify the effectiveness and significance of IMPA. This article achieves significant differences in the distribution of multiple populations by testing the rank of each sample. It shows the average scores obtained from Friedman's tests on benchmark functions for various algorithms. The results show IMPA is the optimal solution in most of the problems and outperforms other algorithms because of the smallest rank mean and standard deviation.

**Figure 4:**
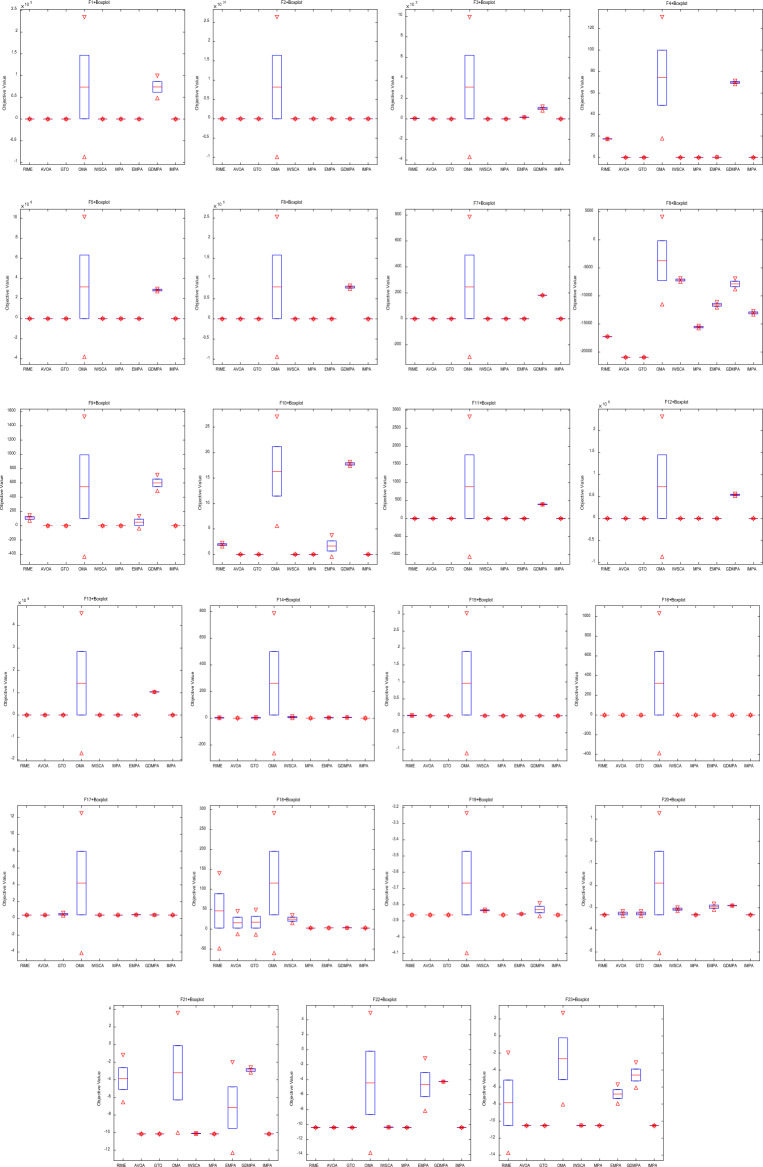
Box-plot for benchmark functions.

In Fig. 4, we present box-plot analysis of the global optimal solutions obtained by the comparison algorithms across 20 independent experiments involving various test functions. This visualization underscores that the IMPA consistently converges to the theoretical extreme values for different test functions, exhibiting accuracy of optimal values and robust algorithm performance. Conversely, results obtained from the other algorithms display significant deviations from the theoretical extreme values, and they exhibit substantial volatility, indicating poor algorithmic robustness. IMPA exhibits better accuracy and stability than other competitive algorithms.

### IEEE CEC2021 experiments

In order to comprehensively assess and compare the performance of the IMPA on Large Scale Global Optimization (LSGO) challenges, we leveraged the IEEE CEC2021 benchmark suite in this research endeavor. This benchmark suite has been meticulously curated to pose intricate optimization problems, as elucidated in Table 4, thus illuminating the multifaceted complexities characterizing the optimization landscapes of these challenges^32,33^. In maintaining methodological consistency, we conducted 20 independent runs on each function, employing similar population sizes and iteration counts as our previous experiments. This stringent approach ensured a robust evaluation of the algorithms under scrutiny.

**Table 4:** CEC2021 functions.

	No	Functions	Dim	F_i_*
Unimodal Function	CEC01	Shifted and Rotated Bent Cigar Function(CEC 2017[4]F1)	20	100
Basic Functions	CEC02	Shifted and Rotated Schwefel’s Function(CEC 2014[3]F11)	20	1100
	CEC03	Shifted and Rotated Lunacek bi-RastriginFunction(CEC 2017[4]F7)	20	700
	CEC04	Expanded Rosenbrock’s plus Griewangk’s Function (CEC2017[4]f19)	20	1900
Hybrid Functions	CEC05	Hybrid Function 1 (N = 3) (CEC 2014[3]F17)	20	1700
	CEC06	Hybrid Function 2 (N = 4) (CEC 2017[4]F16)	20	1600
	CEC07	Hybrid Function 3 (N = 5) (CEC 2014[3]F21)	20	2100
Composition Functions	CEC08	Composition Function1 (N = 3) (CEC 2017[4]F22)	20	2200
	CEC09	Composition Function2 (N = 4) (CEC 2017[4]F24)	20	2400
	CEC10	Composition Function3 (N = 5) (CEC 2017[4]F25)	20	2500

The function values are summarized in Table 5, which provide an in-depth analysis of the performance of the IMPA and its comparison to other algorithms in IEEE CEC2021. It shows that the IMPA and the OMA get closer to the minimum values than other algorithms. Evidently, the IMPA exhibited a noteworthy propensity to approach the minimal values more closely than any other algorithms. Notably, the IMPA excelled in its performance, surpassing other algorithms except the OMA. It demonstrated substantial advancements in accuracy and convergence speed across the majority of CEC2021 functions. The IMPA obtain the remarkable performances, which outperforms other algorithms in solving LSGO problems with a significant improvement in accuracy and convergence speed for most of CEC2021 functions.

**Table 5:** Comparison results for CEC2021 functions.

		**RIME**	**AVOA**	**GTO**	**OMA**	**IWSCA**	**MPA**	**EMPA**	**GDMPA**	**IMPA**
CEC01	Mean	7.26E−15	1.46E−43	5.75E−114	0*	834,963,577	818,502.9403	5.2206E−181	6,634,289	0*
	Std	7.37E−15	2.06245E−43	8.13E−114	0*	279,503,679.3	396,172.0762	0	7,222,330	0*
CEC02	Mean	1.06E+01	9.09E−13	6.41E−05	0*	3879.515682	1021.22229	1.81899E−12	1887.214	0*
	Std	1.44E+01	1.28622E−12	9.07E−05	0*	158.3116298	236.498377	0	233.516	0*
CEC03	Mean	4.84E+00	9.86E−32	1.39E+02	0*	472.3799902	63.7474567	0*	50.95912	0*
	Std	6.85E+00	1.39452E−31	1.57E+01	0*	33.04189021	18.13018351	0*	0.49358	0*
CEC04	Mean	4.48E−02	0*	0*	0*	2959.379557	7.12936811	0*	12.67865	0*
	Std	6.33E−02	0*	0*	0*	1019.36664	0.407156896	0*	2.442481	0*
CEC05	Mean	2.43E+00	2.59E−15	6.12E+02	0*	38,777.18388	1495.320722	4.37878E−21	1027.808	0*
	Std	3.44E+00	3.58619E−15	1.06E+00	0*	52,671.25103	344.1330117	5.95515E−21	291.543	0*
CEC06	Mean	3.48E+00	6.65E−01	3.60E+02	0*	564.9775349	306.3694926	0.00029594	379.8485	0*
	Std	4.55E+00	0.799318612	4.05E+02	0*	127.1071849	85.16297225	1.7579E−05	40.32283	0*
CEC07	Mean	2.02E−01	7.19E−02	2.26E−05	0*	970.7126115	328.3057551	0.00015733	713.1589	0*
	Std	1.18E−01	0.047040421	2.92E−05	0*	54.97858291	204.4821771	6.02264E−05	104.4186	0*
CEC08	Mean	5.29E−14	1.85E−16	0.00E+00	0*	823.977978	1433.530948	0*	1001.808	0*
	Std	1.37E−14	2.61682E−16	0.00E+00	0*	109.4682969	166.4487987	0*	33.19588	0*
CEC09	Mean	6.11E−11	1.33E−14	4.44E−15	0*	301.7061741	9.820447789	8.88178E−15	7.863567	0*
	Std	5.01E−11	6.28037E−15	6.28E−15	0*	223.6835374	1.200687047	0	2.582491	0*
CEC10	Mean	4.35E+01	8.12E−02	3.55E−15	0*	140.0561946	55.37478999	0.001562257	51.1911	0*
	Std	6.16E+01	0.01166407	5.02E−15	0*	24.40969325	2.047167701	8.63846E−05	0.4499	0*

*Represent the optimal solution of the function.

**Figure 5:**
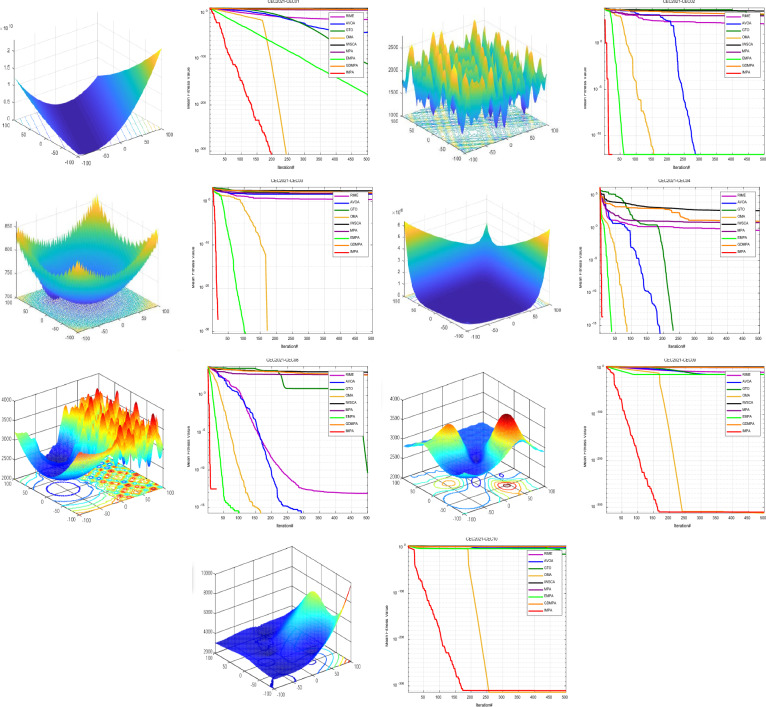
Convergence curves for CEC 2021 functions.

The convergence provide a visual narrative of algorithmic behavior by Fig. 5. The IMPA emerges as a frontrunner, consistently yielding smaller function values, thereby expediting the convergence process. In particular, the IMPA outperformed other algorithms in achieving theoretical global minima within a mere handful of iterations for functions of CEC2021. Additionally, the IMPA exhibited notable celerity in terms of convergence and precision. The IMPA also exhibits faster convergence than other algorithms, reaching the theoretical global minimum within 200 iterations. Even in the context of Composition Functions, the IMPA demonstrates superior convergence speed and accuracy relative to other algorithms, highlighting its distinctive characteristics.

Conversely, the other algorithms consistently experience premature convergence across all benchmark functions, yielding solutions consistently inferior to those of IMPA. Although OMA performs better than IMPA for CEC06-CEC10, they lag in other scenarios. The convergence curves demonstrate that IMPA consistently reaches the theoretical global minimum for most benchmark functions, underscoring its superior optimization performance. The outstanding convergence speed of IMPA significantly reduces the computational complexity of the optimization problem.

Wilcoxon rank-sum test and Friedman test are also introduced to analyze the results of the algorithm for IEEE CEC2021. Wilcoxon rank-sum test results are shown in Table 6, the *p-value* ​​of the IMPA are less than 0.05, which is indicated that there is a significant difference between the IMPA and other comparison algorithms except for GTO and OMA. it is obvious that the IMPA performs better than other algorithms, and the improvement strategy introduced in the IMPA is effective. Friedman test results are shown in Table 7, which can be concluded that the IMPA is competitive when compared with other improvement algorithms as it can get closer to the global optimal solution of the function.

**Table 6:** Wilcoxon rank-sum test.

	**IMPA**									
	CEC01	CEC02	CEC03	CEC04	CEC05	CEC06	CEC07	CEC08	CEC09	CEC10
RIME	+	+	+	+	+	+	+	+	+	+
AVOA	+	+	+	+	NAN	+	+	+	+	+
GTO	−	−	−	−	−	−	−	−	−	−
OMA	−	−	−	NAN	−	−	−	−	−	−
IWSCA	+	+	+	+	−	+	+	+	+	+
MPA	+	+	+	+	+	+	+	+	+	NAN
EMPA	+	+	+	+	+	+	+	+	+	+
GDMPA	+	+	+	+	+	+	+	+	+	+

**Table 7:** Friedman test.

	IMPA	RIME	AVOA	GTO	OMA	IWSCA	MPA	EMPA	GDMPA
CEC01	1.00*	8.23	2.97	4.83	8.77	2.03	4.50	5.83	10.00
CEC02	1.77*	7.63	3.28	5.20	9.60	3.28	3.47	7.10	8.87
CEC03	2.50	9.50	1.60*	9.50	8.00	3.30	4.63	6.00	7.00
CEC04	1.50 *	8.60	3.43	3.43	7.60	3.80	3.70	10.00	7.57
CEC05	1.00*	9.40	3.10	8.03	9.57	5.17	6.83	1.60	2.07
CEC06	3.28	7.87	3.23*	9.77	4.55	6.57	8.40	1.00	2.88
CEC07	1.60*	2.95	3.12	6.80	2.95	5.13	7.07	9.20	4.77
CEC08	3.43	2.43	4.73	5.20	8.20	7.23	3.57	9.90	3.20*
CEC09	3.63	4.13	4.13	4.13	4.13	4.13	7.07	4.13*	4.13*
CEC10	3.80	3.07 *	4.62	9.90	3.07*	5.17	7.10	3.07	3.07 *
**Mean**	2.35*	6.38	3.42	6.68	6.64	4.58	5.63	5.78	5.36
**Std**	1.05*	2.73	0.86	2.34	2.53	1.50	1.74	3.14	2.65

*Represent the optimal solution of the function.

**Figure 6:**
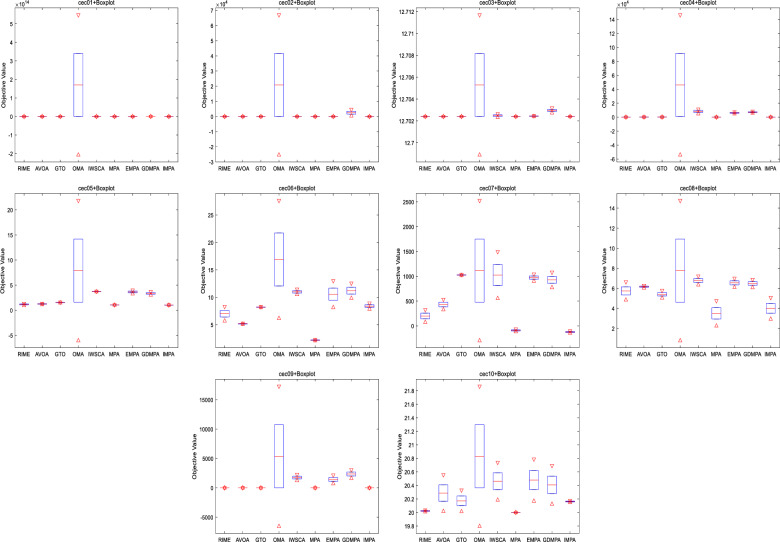
Box-plot for CEC 2021 functions.

Box-plot analysis of global optimal solutions are shown by Fig. 6. The findings elucidate that IMPA consistently converges to the theoretical extreme values of these functions, maintaining stable distributions and exemplifying algorithmic robustness. In contrast, the results obtained from other algorithms are marked by significant deviations from the expected extreme values, indicating a degree of instability and reduced robustness in their algorithmic compositions. IMPA exhibits better accuracy and stability than other competitive algorithms.

Ablation experiments is also introduced to analyze the performance of IMPA. In this study, the improved adaptive weighting MPA (WMPA), the improved dynamic social strategy MPA (SMPA), and the complete improved algorithm IMPA are used to compare the experiments on unimodal (F1–F3), multimodal (F10–F12), and fixed-dimension (F21–F23) test functions, respectively, and the final results are shown in the Table 8.

**Table 8:** Ablation experiments.

		SMPA	WMPA	IMPA
F1	Mean	1.41595E−53	7.33482E−05	0
	Std	1.92364E−53	1.09986E−06	0
F2	Mean	1.41462E−32	0.054758526	0
	Std	1.09613E−32	0.001550533	0
F3	Mean	2.51941E−14	0.000268023	0
	Std	3.54353E−14	0.000130215	0
F10	Mean	4.44089E−15	0.00562099	8.88178E−16
	Std	0	0.000366998	0
F11	Mean	0	2.24666E−06	0
	Std	0	4.34984E−07	0
F12	Mean	0.000103664	0.079703293	5.52733E−11
	Std	4.50088E−06	0.019973575	7.00712E−11
F21	Mean	−10.15319968	−10.15319968	−10.1532
	Std	1.77636E−15	0	1.78E−15
F22	Mean	−10.40294057	−10.40294057	−10.4029
	Std	0	0	0
F23	Mean	−10.53640982	−10.53640982	−10.5364
	Std	0	0	3.97E−15

SMPA can be close to the optimal value in all three types of test functions, which can improve the performance of the MPA algorithm, while WMPA can only achieve excellent results in the fixed dimension function test, and can not achieve the optimal solution in the unimodal and multimodal test functions. Compared with the combination of the two strategies, IMPA has better performance and effectively solves the problem of the algorithm falling into local optimum.

## Engineering design optimization

Mechanical optimization problems are inherently entwined with mathematical modeling. The key of constructing an optimal design mathematical model lies in the identification of design variables, objective functions, and constraints. In our quest to assess the efficacy of the newly proposed IMPA, we have turned our focus to a selection of real-world industrial engineering design challenges. These problems are enriched with constraints that reflect practical engineering scenarios. We present these challenges below, as they form the basis of the experimentation phase.

### Welded beam design

**Figure 7:**
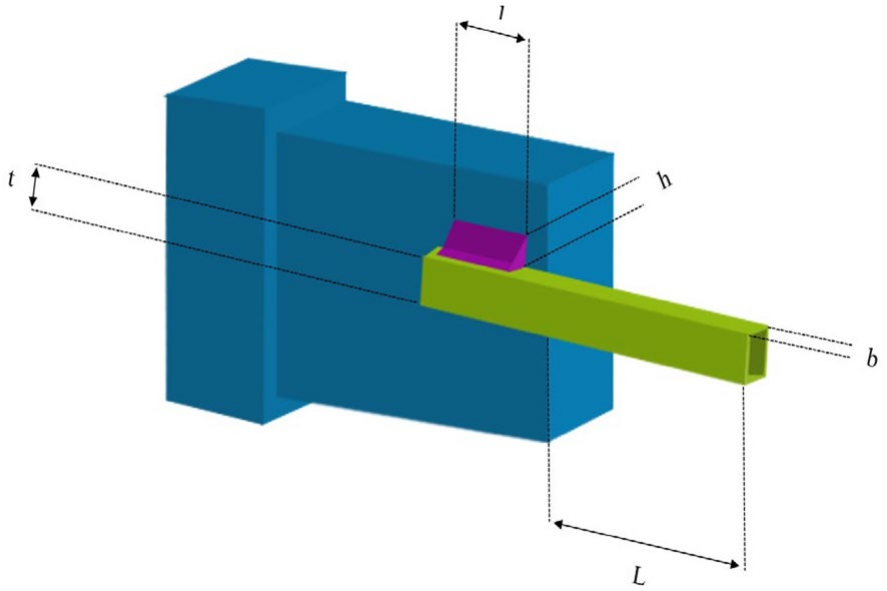
Welded Beam Design.

The objective of this particular engineering challenge revolves around the minimization of the weight of a welded beam^34^. The core mission in this problem is the optimization of these four primary parameters, seeking to minimize the weight of the welded beam. These parameters are articulated as the width of the welded (*h*), the height of the clamped bar (*t*), the length of the bar (*l*), and the width of the bar (*b*), whose interplay illustrated in Fig. 7. The mathematical model of welding beam design is as follows:

$$\begin{gathered} x = (x_{1} ,x_{2} ,x_{3} ,x_{4} ) = (h,t,l,b) \hfill \\ g_{1} (x) = \tau (X) - 13600 \le 0 \hfill \\ g_{2} (x) = \sigma (X) - 36000 \le 0 \hfill \\ g_{3} (x) = x_{1} - x_{4} \le 0 \hfill \\ g_{4} (x) = 0.1047x_{1}^{2} + 0.04811x_{3} x_{4} (x_{2} + 14) - 5 \le 0 \hfill \\ g_{5} (x) = 0.125 - x_{1} \le 0 \hfill \\ g_{6} (x) = \delta (X) - 0.25_{1} \le 0 \hfill \\ g_{7} (x) = 6000 - P_{c} (x) \le 0 \hfill \\ f(x) = 1.10471x_{1}^{2} x_{2} + 0.04811x_{3} x_{4} (14 + x_{2} ) \hfill \\ \end{gathered}$$

The results stemming from the application of the proposed IMPA are diligently compared with those obtained through other established methods. The outcomes are meticulously detailed in Table 9, providing optimal parameter values and objective function values for all the algorithms under comparative scrutiny. Remarkably, the IMPA stands out as a paragon of reliability, outperforming other state-of-the-art methodologies. It yields optimal variables represented as *h* = *2.42253E−01, l* = *6.15443E*+*00, t* = *8.54323E*+*00, b* = *2.64534E−01*, accompanied by the best objective function value *f(x)* = *1.69524E*+*00*. The overall average are also superior to other algorithms, indicating that the IMPA algorithm proposed in this paper exhibits excellent optimization ability and stability for the design problem.

**Table 9:** Comparison results for Welded Beam Design.

**Algorithm**	*h*	*l*	*t*	*b*	*f(x)*
RIME	2.23490E−01	6.23300E+00	8.43230E+00	2.43215E−−01	5.57000E+08
AVOA	2.43500E−01	6.32400E+00	8.43220E+00	2.54344E−01	1.79000E+00
GTO	2.54343E−01	6.54342E+00	8.54353E+00	2.43252E−01	1.05093E+05
OMA	3.14332E−01	7.43252E+00	8.13230E+00	2.23445E−01	3.30139E+17
IWSCA	2.48900E−01	6.17320E+00	8.51220E+00	3.21215E−01	3.12110E+00
MPA	2.54300E−01	7.32300E+00	9.13800E+00	2.54315E−01	1.70000E+00
EMPA	2.34520E−01	6.53300E+00	7.13200E+00	2.25315E−01	3.89153E+00
GDMPA	2.43250E−01	6.34300E+00	8.54432E+00	2.54323E−01	4.44063E+00
IMPA	2.42253E−01	6.15443E+00	8.54323E+00	2.64534E−01	1.69524E+00

**Figure 8:**
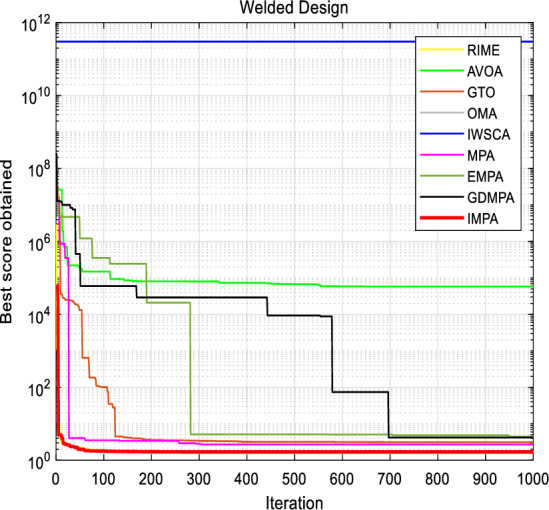
Convergence curves for Welded Beam Design.

For a visual insight into the effectiveness of the IMPA, the objective function is presented in Fig. 8. This graphical representations aptly showcase the IMPA's ability to swiftly converge towards the optimal solution within 15 iterations. The parameter values and parameter's curve further substantiate the algorithm's efficacy. It is abundantly clear that the recommended IMPA has delivered outstanding results, yielding optimal parameters and objective function values that contribute significantly to the problem's resolution.

### Tension/compression spring design

**Figure 9:**
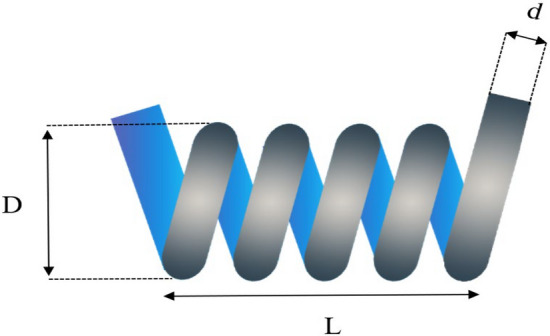
Tension/Compression Spring Design.

The primary objective of this study revolves around the reduction of total weight in a specific spring design problem^35^. The fundamental of this tension/compression spring design issue lies in the quest to minimize the overall weight of the designated spring. This endeavor necessitates the modulation of three key variables, namely wire diameter (*d*), mean coil diameter (*D*), and the number of active coils (*N*), as visually portrayed in Fig. 9. The mathematical model of tension/compression spring design is described as follows:

$$\begin{aligned} x & = (x_{1} ,x_{2} ,x_{3} ) = (h,D,N) \\ g_{1} (x) & = 1 - \frac{{x_{2}^{2} x_{3} }}{{71785x_{1}^{4} }} \le 0 \\ g_{2} (x) & = \frac{{4x_{2}^{2} - x_{1} x_{2} }}{{12566(x_{1}^{3} x_{2} - x_{1}^{4} )}} - \frac{1}{{5108x_{1}^{2} }} - 1 \le 0 \\ g_{3} (x) & = 1 - \frac{{140.45x_{1} }}{{x_{2}^{2} x_{3} }} \le 0 \\ g_{4} (x) & = \frac{{x_{1} + x_{2} }}{1.5} - 1 \le 0 \\ f(x) & = x_{1}^{2} x_{2} (2 + x_{3} ) \le 0 \\ \end{aligned}$$

The IMPA performance is assessed in comparison to other established methods. Table 10 presents the results and optimal parameter values, encompassing the best outcomes for all comparative techniques, including the proposed IMPA. Remarkably, the IMPA showcases superior performance, furnishing a more robust solution with optimal variable values at *d* = *5.17750E−02, D* = *3.58791E−01, N* = *1.11683E*+*01*, and the best objective value of *f(x)* = *3.63877E*+*00.* The overall average are also superior to other algorithms, indicating that the IMPA algorithm proposed in this paper exhibits excellent optimization ability and stability for the design problem.

**Table 10:** Comparison results for Tension/Compression Spring Design.

**Algorithm**	*d*	*D*	*N*	*f(x)*
RIME	5.16788E−02	3.56874E−01	1.12568E+01	4.78000E+02
AVOA	5.19874E−02	3.68742E−01	1.35488E+01	5.15000E+01
GTO	5.25468E−02	3.65440E−01	1.23154E+01	5.18679E+01
OMA	5.18798E−02	3.87890E−01	1.45475E+01	1.53519E+04
IWSCA	5.18698E−02	3.35409E−01	1.36897E+01	4.44719E+01
MPA	5.16430E−02	3.36300E−01	1.13959E+01	3.64000E+00
EMPA	5.19432E−02	3.39781E−01	1.68896E+01	6.64441E+01
GDMPA	5.18868E−02	3.43530E−01	1.58759E+01	4.92905E+01
IMPA	5.17750E−02	3.58791E−01	1.11683E+01	3.63877E+00

**Figure 10:**
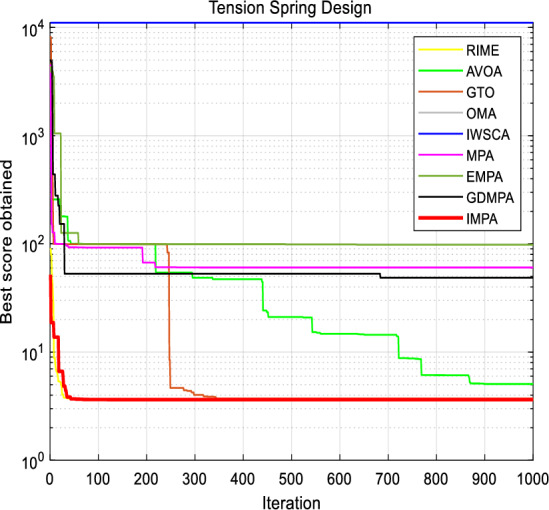
Convergence curves for Tension Spring Design.

The objective cost curves of the proposed IMPA, which are presented in Fig. 10. This graphical representations offer a compelling illustration of the IMPA's ability to swiftly converge towards the optimal solution within the first 20 iterations. The curves of the parameter values further underscore the efficacy of the IMPA.

### Pressure vessel design

**Figure 11:**
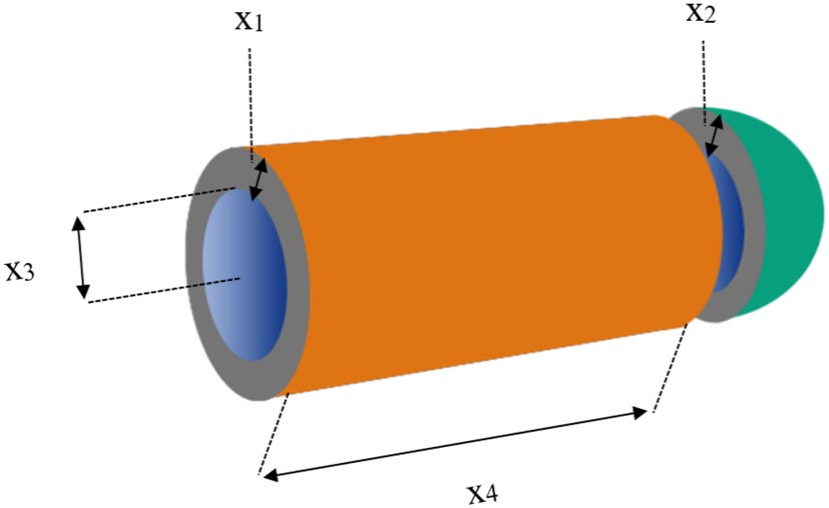
Pressure Vessel Design.

The primary aim of this engineering challenge is to minimize the weight of a specific type of cylindrical pressure vessel^36^. This study centers its examination on four key design parameters, which include the shell width (*x*_*1*_), head width (*x*_*2*_), internal radius (*x*_*3*_), and the height of the cylindrical part, excluding the head (*x*_*4*_), as thoughtfully illustrated in Fig. 11. The mathematical model of pressure vessel design is described as follows:

$$\begin{aligned} x & = (x_{1} ,x_{2} ,x_{3} ,x_{4} ) = (T_{s} ,T_{h} ,R,L) \\ g_{1} (x) & = 0.0193x_{3} - x_{1} \le 0 \\ g_{2} (x) & = 0.00954x_{3} - x_{2} \le 0 \\ g_{3} (x) & = 1296000 - 4/3\pi x_{3}^{3} - \pi x_{3}^{2} x_{4} \le 0 \\ g_{4} (x) & = x_{4} - 240 \le 0 \\ f(x) & = 0.6224x_{1} x_{3} x{\phantom{0}}_{4} + 1.7781x_{2} x_{3}^{2} + 19.84x_{1}^{2} x_{3} + 3.1661x_{1}^{2} x_{4} \\ \end{aligned}$$

IMPA's results are meticulously assessed and juxtaposed with those obtained through other well-established techniques. A comprehensive presentation of the results is encapsulated in Table 11, featuring optimal parameter values achieved by each algorithm. The proposed IMPA emerges as a beacon of reliability, yielding optimal parameters represented as *x*_*1*_ = *8.125000E−01, x*_*2*_ = *4.37500E−01, x*_*3*_ = *4.209840E*+*01 and x*_*4*_ = *1.76638E*+*03,* along with the best objective value *f(x)* = *2.49878E*+*03*. The overall average are also superior to other algorithms, indicating that the IMPA algorithm proposed in this paper exhibits excellent optimization ability and stability for the design problem.

**Table 11:** Comparison results for Pressure Vessel Design.

Algorithm	*x*_*1*_	*x*_*2*_	*x*_*3*_	*x*_*4*_	*f(x)*
RIME	8.125000E−01	4.37500E−01	4.204861E+01	1.77707E+03	3.13000E+03
AVOA	8.250000E−01	6.25000E−01	5.598700E+01	1.84454E+03	2.50000E+03
GTO	9.375000E−01	4.37500E−01	4.209130E+01	1.76746E+03	2.49899E+03
OMA	8.125000E−01	4.34500E−01	4.032390E+01	1.12679E+03	9.29135E+08
IWSCA	9.375000E−01	5.00000E−01	4.832900E+01	1.17711E+03	2.56228E+03
MPA	8.125000E−01	4.37500E−01	4.206500E+01	1.80823E+03	2.50000E+03
EMPA	8.125000E−01	4.12500E−01	4.209130E+01	1.76746E+03	2.57532E+03
GDMPA	7.975000E−01	5.22000E−01	4.132390E+01	1.52123E+03	2.59322E+03
IMPA	8.125000E−01	4.37500E−01	4.209840E+01	1.76638E+03	2.49878E+03

**Figure 12:**
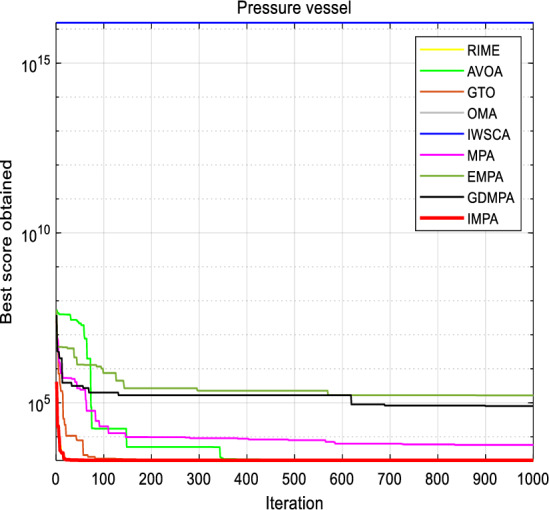
Convergence curves for Pressure Vessel Design.

To substantiate the algorithm's efficacy, the objective cost curves is presented in Fig. 12. These visual representations aptly demonstrate that the IMPA swiftly converged to the optimal solution before reaching the 30th iteration. The figures displaying parameter values and the curve further reinforce the algorithm's effectiveness in addressing the pressure vessel design problem.

### Three bar design

This question is intended to minimize the weight of a particular three shot Truss^37^. The two main design parameters studied in this issue are the cross-sectional area of component 1 (*x*_1_), component 2 (*x*_2_) and component 3 (*x*_3_), as shown in Fig. 12. Use the proposed IMPA to obtain the optimal solution and compare the results with other established methods. The mathematical model of the Three-bar truss design problem is mathematically described as follows:

$$\begin{aligned} x & = (x_{1} ,x_{2} ) = (S_{1} S_{2} ) \\ f(x) & = (2\sqrt 2 x_{1} + x_{2} ) \times l \\ R_{1} (x) & = \frac{{\sqrt 2 x_{1} + x_{2} }}{{\sqrt 2 x_{1}^{2} + 2x_{1} x_{2} }}P - \varphi \le 0 \\ R_{2} (x) & = \frac{{x_{2} }}{{\sqrt 2 x_{1}^{2} + 2x_{1} x_{2} }}P - \varphi \le 0 \\ R_{3} (x) & = \frac{{x_{2} }}{{\sqrt 2 x_{1} + x_{2} }}P - \varphi \le 0 \\ f(x) & = (2\sqrt 2 x_{1} + x_{2} ) \times l \\ l & = 100cm,P = 2KN/cm,\varphi = 2KN/cm \\ \end{aligned}$$

Table 12 summarizes the results of the competitive approach in solving the three link Truss design problem, including the recommended IMPA. This table provides the optimal parameter values obtained by all algorithms. It is evident that the proposed IMPA produces more reliable results than other state-of-the-art methods. The optimal solution obtained by IMPA is *x*_*1*_ = *7.886654E−01 and x*_*2*_ = *4.082758E−0*1, with the optimal objective function value *f(x)* = *1.863895E*+*02*. The overall average are also superior to other algorithms, indicating that the IMPA algorithm proposed in this paper exhibits excellent optimization ability and stability for the design problem.

**Table 12:** Comparison results for Three Bar Truss Design.

Algorithm	*x*_*1*_	*x*_*2*_	*f(x)*
RIME	7.88249E−01	4.07589E−01	2.69000E+03
AVOA	7.87588E−01	4.15789E−01	1.87000E+02
GTO	7.85787E−01	4.12234E−01	1.92644E+02
OMA	7.97575E−01	4.09634E−01	5.64050E+03
IWSCA	7.96758E−01	4.09638E−01	1.86949E+02
MPA	7.88649E−01	4.08234E−01	1.86000E+02
EMPA	7.33575E−01	4.09834E−01	1.86719E+02
GDMPA	7.86685E−01	4.09634E−01	1.86442E+02
IMPA	7.88665E−01	4.08275E−01	1.86389E+02

**Figure 13:**
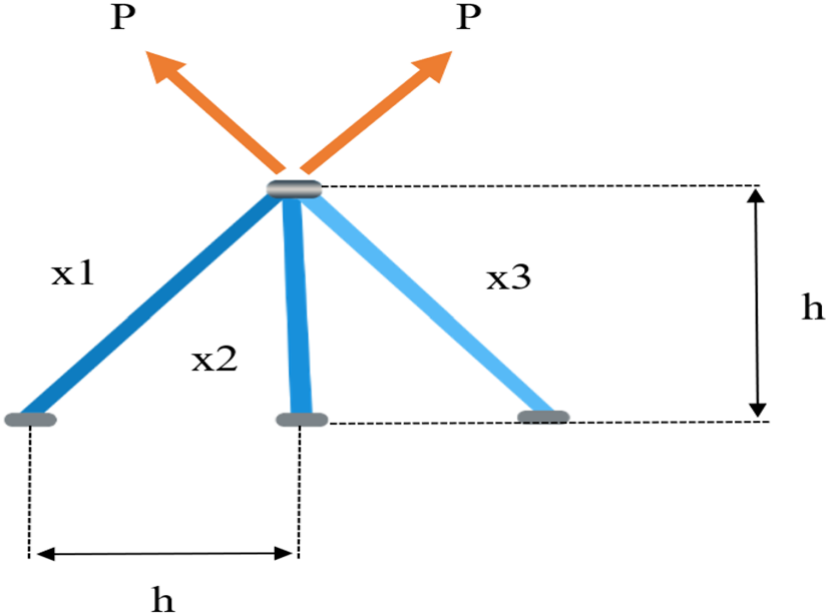
Three Bar Truss Design.

**Figure 14:**
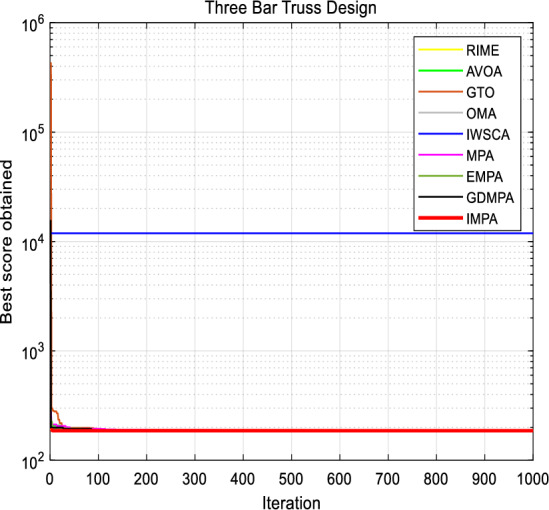
Convergence curves for Three Bar Truss.

Figure 13 shows the convergence curve of the objective function of the proposed IMPA. The IMPA can quickly find the optimal solution, which can be achieved with only 15 iterations, as shown in the convergence diagram in Fig. 14. The first parameter curve aim to demonstrate the effectiveness of the proposed IMPA. The convergence curve indicates that the IMPA provides excellent results and quickly provides an ideal solution. In addition, the diversity of potential solutions has been confirmed.

## Conclusion

In summary, the IMPA is a novel optimization algorithm for solving industrial engineering design problems. The IMPA is an innovative approach to the Marine Predators Algorithm by incorporating dynamic social strategy and self-adaptive weight setting, which can relatively quickly find near-optimal solutions and overcome the limitations by avoiding local optima and improving overall performance. The IMPA contributes to the balance of exploration and exploitation through dynamic social strategy and self-adaptive weight. The superiority of IMPA in CEC 2021 functions has been demonstrated. Meanwhile the results of this study demonstrate the effectiveness of the IMPA in finding high-quality solutions for industrial engineering design problems. Compared with optimization methods and other versions of MPA, including versions of MPA, EMPA, GDMPA, RIME, AVOA, GTO, OMA, IWSCA, the IMPA has several advantages and faster convergence to the optimal solution and reduced chances of falling into local minima. Box-plot analyses prove that the IMPA has better accuracy and convergence than other algorithms. Wilcoxon rank tests which performed the method also show that IMPA differs significantly from any other algorithm. The IMPA has also been proven to be more effective in finding high-quality solutions in a shorter period of time. In order to provide a promising solution for industrial engineering design problems and highlight the potential of the IMPA as a useful tool for solving real-world problems. This study has implemented four highly representative engineering design problems, including Welded Beam Design, Tension/Compression Spring Design, Pressure Vessel Design, and Three Bar Design. The experimental results also proved its efficiency in successfully solving complex industrial engineering design problems.

Every method has advantages and disadvantages, and the IMPA is no exception. The improved algorithm may be performed worse than the GTO and OMA for IEEE CEC2021. Therefore in further investigation, we may combine all chaos mapping in the same algorithm and use some adaptive strategies to decide which parameters will be activated. Future research should focus on implementing IMPA on multi-objective optimization. This would allow for more extensive testing of the algorithm's capabilities and provide a better understanding of its potential in real-world applications.

## Data Availability

Data used in this study are available upon reasonable request to the corresponding author.
